# NUT midline carcinoma mimicking a germ cell tumor: a case report

**DOI:** 10.1186/s12885-016-2944-3

**Published:** 2016-11-17

**Authors:** Yohei Harada, Takafumi Koyama, Kengo Takeuchi, Kazufusa Shoji, Kazuei Hoshi, Yu Oyama

**Affiliations:** 1 Department of Medical Oncology, Kameda Medical Center, Kamogawa, Chiba 296-8602 Japan; 2 Department of Internal Medicine, Division of Haematology, Respiratory Medicine and Oncology, Faculty of Medicine, Saga University, Saga, Japan; 3 Pathology Project for Molecular Targets, the Cancer Institute, Japanese Foundation for Cancer Research, Tokyo, Japan; 4 Division of Pathology, the Cancer Institute, Japanese Foundation for Cancer Research, Tokyo, Japan; 5 Department of Radiation Therapy, Kameda Medical Center, Kamogawa, Chiba 296-8602 Japan; 6 Department of Diagnostic Pathology, Kameda Medical Center, Kamogawa, Chiba 296-8602 Japan

**Keywords:** NUT midline carcinoma, Tumor markers, Alpha-fetoprotein, Radiotherapy, PET/CT, Case report

## Abstract

**Background:**

NUT midline carcinoma (NMC) is a rare and highly aggressive malignancy. Although more information on NMC has been recently accumulating in the literature, most oncologists and pathologists remain unfamiliar with the clinical and pathologic features of this disease. The clinical features of NMC sometimes mimic those of other malignancies, and NMC can therefore be overlooked if the diagnosis is not suspected. We present the case of a young male with NMC arising in the mediastinum with elevated serum alpha-fetoprotein levels suggestive of an extragonadal nonseminomatous germ-cell tumor.

**Case presentation:**

A 28-year-old Japanese male presented with cough and left-sided chest pain for 6 weeks. The patient had a mediastinal tumor with metastases to the right lung, lymph nodes, and bones at initial presentation. Nonseminomatous germ cell tumor was suspected due to the young age, location of the tumors, and elevated serum alpha-fetoprotein. However, biopsy confirmed the diagnosis of NMC with immunohistochemistry. The tumor briefly responded to cytotoxic chemotherapy but subsequently progressed and became refractory to the chemotherapy regimen. External beam radiotherapy was administered with dramatic shrinkage of the tumor and a metabolic response on 18-fluoro-2-deoxyglucose positron emission tomography/computed tomography (^18^F-FDG PET/CT) scan. However, the patient died 4.5 months after the diagnosis of NMC.

**Conclusions:**

Serum levels of alpha-fetoprotein may be elevated in patients with NMC. Regardless of the level of tumor markers, immunohistochemistry for NUT should be performed in cases of poorly differentiated carcinomas without glandular differentiation arising in the midline structures. ^18^F-FDG PET/CT is useful for staging and assessing responses to therapy.

## Background

NUT midline carcinoma (NMC) is a highly aggressive subset of squamous cell carcinomas, affecting both children and adults [1]. The genetic hallmark is a rearrangement of the *NUT* gene, located on chromosome 15 [2]. The rearrangement commonly occurs between the *NUT* gene and *BET* family genes *BRD4* and *BRD3* [1], although other rare fusion partners of the *NUT* gene have also been recently reported [3].

Because of the poor prognosis (median survival 6.7 months) [2] and poor response to conventional cytotoxic chemotherapy, new drugs such as BET inhibitor (BETi) and histone deacetylase inhibitor (HDACi) are now in clinical trials for patients with NMC [3]. Because of the availability of these potentially promising new investigational drugs, prompt diagnosis of NMC is even more important to plan appropriate treatment and to encourage patients to consider participating in clinical trials. Most oncologists and pathologists are not familiar with NMC owing to its rarity. The clinical features of NMC sometimes mimic those of other malignancies. For these reasons, NMC may often be misdiagnosed if it is not suspected and specifically looked for. In one study, 114 cases of poorly differentiated carcinomas or unclassified mediastinal malignancies were pathologically reexamined using immunohistochemistry for NUT and fluorescence in situ hybridization (FISH), leading to the diagnosis of NMC in 4 (3.5%) cases [4]. Here we report the case of a young male with NMC arising in the mediastinum with elevated serum alpha-fetoprotein (AFP) levels, suggestive of an extra-gonadal nonseminomatous germ cell tumor (NSGCT).

## Case presentation

**Fig. 1:**
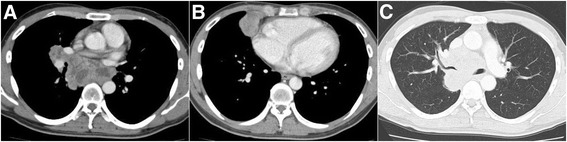
Full-body CT scan at the first visit. Tumor in the mediastinum and lymphadenopathy in the right side of the hilum and supraclavicular region (**a**). A tumor in the right middle lobe (**b**). Right bronchial stenosis due to the mediastinal tumor is shown (**c**)

**Fig. 2:**
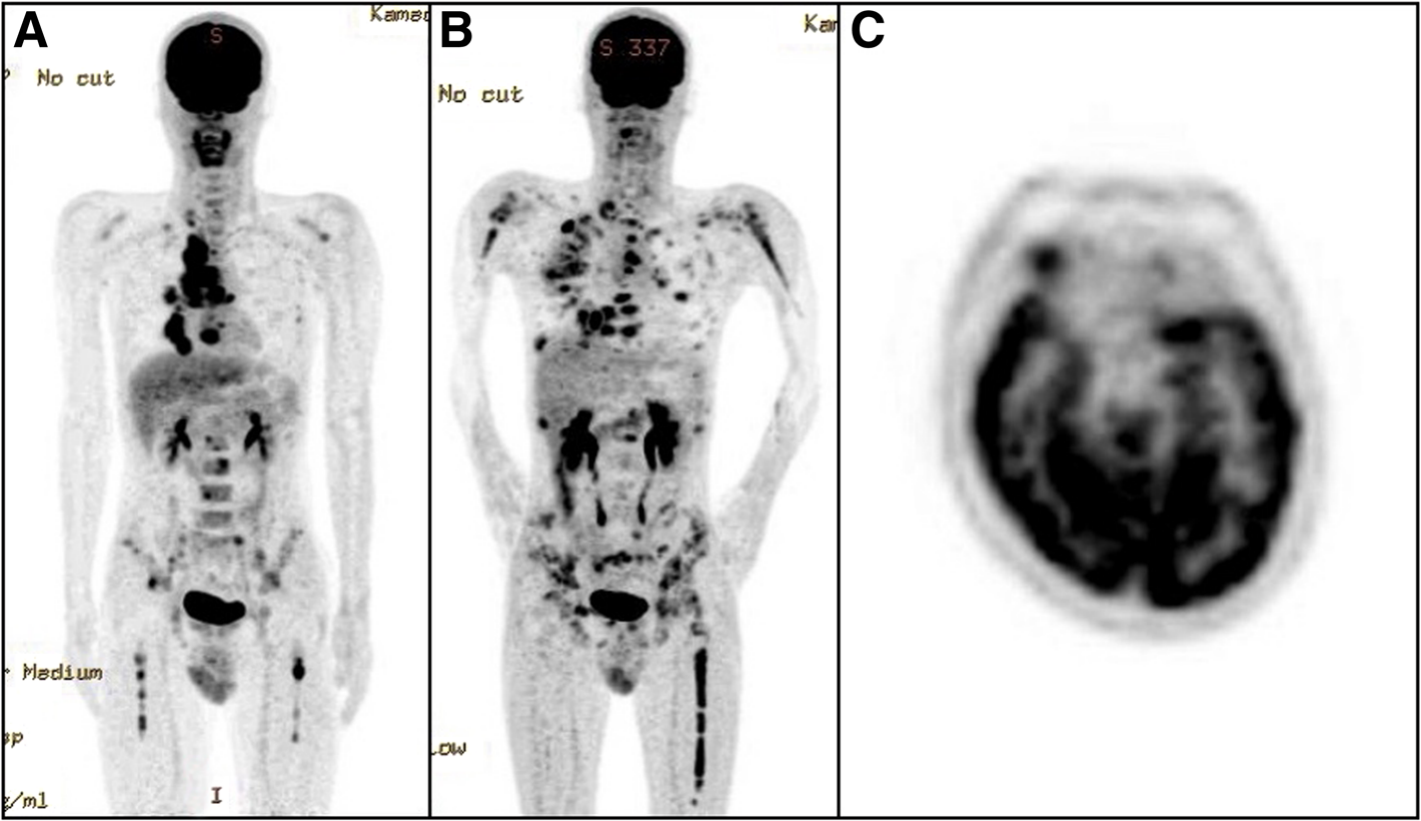
**a** ^18^F-FDG PET/CT scan before chemotherapy, with abnormal FDG uptake seen in the mediastinal tumor and the right lung metastasis, lymph nodes, and multiple bones (spine, scapula, ribs, sternum, pelvis, and femur). **b** ^18^F-FDG PET/CT scan after chemotherapy and radiotherapy to the mediastinum, vertebrae L3–S1, and right femur, showing dramatic shrinkage of the tumor and improved metabolic response, while many new abnormal FDG accumulations are demonstrated in other sites. **c** Abnormal FDG uptake in a mass in the right orbital soft tissues, suggestive of orbital metastasis

A 28-year-old Japanese male presented with cough and left-sided chest pain for 6 weeks. The medical, surgical, and family histories were unremarkable. He smoked approximately 20 cigarettes per day for 6 years and infrequently consumed small amounts of alcohol. Physical examination was unremarkable; the lungs were clear to auscultation. Chest X-ray revealed an enlarged mediastinum. A full-body CT scan showed a bulky mediastinal mass with right bronchial stenosis, lymphadenopathy in the right side of the hilum and supraclavicular region, and a mass in the right middle lobe measuring 4.4 × 3.0 cm (Fig. 1). ^18^F-FDG PET/CT showed the involvement of multiple bones, including spine, scapula, ribs, sternum, pelvis, and femur (Fig. 2a).

The clinical course and patient background suggested a differential diagnosis that included lung cancer, lymphoma, and a mediastinal germ cell tumor (GCT). Laboratory investigations were significant for an elevated serum lactate dehydrogenase [LDH; 667 IU/L (normal range: 119–229 IU/L)], C-reactive protein [0.82 mg/dL (0.01–0.4 mg/dL)], soluble IL-2 receptor [770 U/mL (112–496 U/mL)], and AFP [163.8 ng/mL (0–20 ng/mL)]. Serum levels of β-human chorionic gonadotropin (β-hCG), carcinoembryonic antigen, pro-gastrin-releasing peptide, and cytokeratin-19 fragments (CYFRA) were within normal limits.

**Fig. 3:**
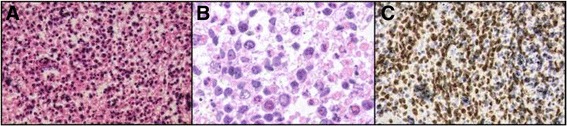
**a** Tumor section (H&E, 100×) with loosely arranged cells with evident necrosis and degeneration and no clear pattern of differentiation. **b** The tumor is composed of ovoid and spindle-shaped cells with anisocytosis, scanty cytoplasm, and irregular ovoid hyperchromatic nuclei. **c** Immunohistochemistry of tumor cell nuclei showing speckled staining for NUT

Pathology examination of tissue from an endobronchial ultrasound-guided transbronchial needle aspiration (EBUS-TBNA) biopsy of a mediastinal lymph node revealed a loosely cohesive growth pattern with prominent necrosis and degeneration and no clear pattern of differentiation (Fig. 3a). The tumor was composed of ovoid and spindle-shaped cells with anisocytosis, scanty cytoplasm, and irregular ovoid hyperchromatic nuclei (Fig. 3b).

Only a minor proportion (<5%) of the cells were positive for AE1/AE3. Squamous cell markers (p40 and p63) were weakly positive in 10–20% of the cells. Neuroendocrine markers (chromogranin and synaptophysin), leukocyte common antigen, myogenic markers (MyoD1 and myogenin), germ cell markers (placental alkaline phosphatase and hCG), c-kit, TTF-1, CA19-9, and CD30 were all negative. The surface markers of tumor cells obtained with flow cytometry were not compatible with lymphoma. A bone marrow aspiration and biopsy revealed infiltration of cells with pathologic features similar to those of the EBUS-TBNA biopsy specimen.

The clinical presentation of a mediastinal tumor in a young male with an elevated serum AFP suggested NSGCT, but findings of immunohistochemistry of tumor sections were not consistent with that diagnosis. An outside pathology consultation was obtained, and the diagnosis of NMC was suggested due to the pathologic and clinical characteristics. Thus, immunohistochemistry using NUT (C52B1) rabbit monoclonal antibody was performed.

While waiting for results of the immunohistochemical examination for NUT, serum levels of LDH increased to 993 IU/L on day 11, suggesting rapid disease progression. Because of the poorly differentiated pattern on histologic analysis, elevated serum AFP, and case reports indicating that cisplatin-based treatment showed some efficacy for treating NMC [5], we decided to start chemotherapy with BEP regimen (bleomycin 30 kU on days 1, 8, and 15; etoposide, 100 mg/m^2^ on days 1–5; and cisplatin, 20 mg/m^2^ on days 1–5; given every 21 days). On day 8 of the first cycle, the immunohistochemistry result revealed that most of the neoplastic cell nuclei were strongly positive for NUT in a speckled pattern (Fig. 3c). The diagnosis of NMC was thus confirmed. After two cycles of chemotherapy, CT showed tumor regression, and serum levels of LDH declined. Therefore, the BEP regimen was continued. However, after three cycles, CT showed tumor progression.

Because the performance status of the patient had declined, we considered single-agent chemotherapy to be most appropriate and started doxorubicin (75 mg/m^2^ every 21 days). Despite the change in chemotherapy regimen, serum levels of LDH continued to increase. Therefore, we judged doxorubicin to be ineffective, although CT scan after the first cycle of doxorubicin showed no change in tumor volume. We believed that local control of the mediastinal mass was most important for the patient at that point to prevent airway obstruction as the tumor progressed. Some authors have reported on the effectiveness of radiotherapy and chemoradiotherapy for NMC [2, 6], we therefore administered mediastinal radiotherapy with concomitant weekly docetaxel (30 mg/m^2^). Radiotherapy was planned with conventional fractionation, 60 Gy/30 fractions (fr).

CT after 16 Gy had been administered showed an apparent decrease in tumor bulk in the irradiated area, although it had increased in other areas. Docetaxel did not seem to be beneficial for systemic tumor control, and platelet counts had decreased by 2.9 × 10^3^/μL; thus, docetaxel was discontinued after four cycles and radiotherapy alone was continued. The patient started to complain of pain in the lower back and right femur; MRI confirmed the presence of osteolytic bone metastases. Palliative radiotherapy (30 Gy/10 fr) for metastases in vertebrae L3–S1 and the right femur was concurrently started with irradiation to the mediastinum. Although the pain in the lower back and right femur were relieved, the patient developed painless proptosis in the right eye. While considering additional radiotherapy to prevent pain, we performed ^18^F-FDG PET/CT to evaluate the extent of metastases to the bones and other organs. It showed that radiotherapy had achieved good local control in the mediastinum, vertebrae L3–S1, and right femur, but there were many new sites of abnormal FDG accumulation (Fig. 2b). Moreover, there was an abnormal FDG uptake in a mass in the right orbital soft tissue (Fig. 2c), suggestive of orbital metastasis. Since then, the patient condition gradually deteriorated, and only palliative care was given. He died 4.5 months after the initial diagnosis of NMC.

## Discussion

Except for the inconsistency with histologic results, the characteristics of the present case for the most part resembled those of extra-gonadal NSGCT: 1) occurrence in young adults, mostly males; 2) midline location; 3) metastases to the lungs, liver, and bones; and 4) elevated serum tumor markers (AFP and hCG) [7].

In an international analysis of mediastinal nonseminomas, an elevated serum AFP was present in 74% (211/287) and β-hCG in 38% (110/287) of the cases [8]. The serum AFP level of the present case was compatible with those results. Aside from the present case, there are only three case reports of NMC with elevated serum AFP levels. In one case, the AFP level was 326 μg/L and β-hCG was <1 IU/L [5]; in another case, they were 62 ng/mL and N/A [9]; in the other, they were 1742 ng/mL and <2 IU/L, respectively [10]. If immunohistochemistry for NUT had not been performed, these cases may have been classified as poorly differentiated carcinoma with midline distribution (extra-gonadal germ cell syndrome), one of the groups of carcinoma of unknown primary with favorable prognosis [11].

French et al. [11] recommended that immunohistochemistry for NUT should be performed in all poorly differentiated carcinomas without glandular differentiation arising in the chest, head, and neck. This means that even if serum tumor markers for GCT are elevated in cases of a tumor arising from such sites, NMC should be suspected.

Definitive diagnosis can be made only by demonstration of nuclear staining using the rabbit monoclonal antibody (clone C52B1) for the NUT protein, even without confirmation of the fusion oncogene with FISH. The antibody is highly sensitive (87%) and specific (100%) in non-GCTs [12]. Because we did not suspect NMC initially and did not cryopreserve the pathology specimen, we could not perform the additional molecular analyses such as FISH. It is noteworthy that GCTs can also display nuclear NUT reactivity, but the staining is very focal (<5% of tumor cells) and faint, and it lacks the speckled pattern [13]. In one report, CD34 immunoreactivity was positive in 7 of 11 NMC cases despite they were epithelial tumor [14]. We performed an additional immunohistological staining of CD34 in the present case after the patient’s death however, no significant CD34 staining was observed.

Another remarkable feature in the present case was the metastasis to the orbital soft tissues. Only four patients with NMC who had orbital involvement have been reported [2, 15–17]. Although involvement of the mediastinum, paranasal sinuses, nasal cavity, intra-thoracic organs, bone, and lymph nodes have been commonly reported, intra-abdominal organs [5, 18] and cutaneous tissue [19] are rare metastatic sites. Till date, there has been no report of brain involvement [20].

The usefulness of ^18^F-FDG PET/CT has been demonstrated in staging and assessing the response to treatment of NMC [21, 22]. In our patient, ^18^F-FDG PET/CT was performed before and after treatment, showing a good response to radiation therapy by the tumor. Metastasis to the bone marrow was shown with ^18^F-FDG PET/CT, but not with CT alone. We therefore recommend performing ^18^F-FDG PET/CT before treatment for accurate staging of NMC.

As in other reports, the tumor in the present case briefly responded to cytotoxic chemotherapy but became refractory to the treatment soon after. There are no specific effective chemotherapeutic regimens [2] because even dose-dense chemotherapy was not found to control the tumor for long [23]. It is probably impossible to cure NMC with cytotoxic chemotherapy alone. Thus, novel targeted therapeutic approaches such as BETi (direct acting inhibitors of the BRD3 and BRD4 bromodomains) and HDACi are highly anticipated [3]. Several phase I clinical trials of BETi (GSK-525762A and OTX015) and HDACi (CUDC-907) are available to patients with NMC. Because of rapid tumor progression and only a short-term response to cytotoxic chemotherapy, we should encourage patients diagnosed with NMC to consider participating in clinical trials as early as possible.

## Conclusions

In summary, we present the case of a patient who had clinical features similar to those of extra-gonadal NSGCT. As serum levels of AFP can be elevated in NMC, immunohistochemistry for NUT should be considered in all poorly differentiated carcinomas arising in midline structures without glandular differentiation, regardless of the levels of tumor markers. ^18^F-FDG PET/CT is useful for staging and assessing the response to therapy. It is expected that novel targeted therapies may change the poor prognosis of NMC in the near future.

## Abbreviations

AFP: Alpha-fetoprotein
BETi: BET inhibitor
EBRT: External beam radiotherapy
EBUS-TBNA: Endobronchial ultrasound-guided transbronchial needle aspiration
FISH: Fluorescence in situ hybridization
fr: fractions
GCT: Germ cell tumor
HDACi: HDAC inhibitor
LDH: Lactate dehydrogenase
NMC: NUT midline carcinoma
NSGCT: Nonseminomatous germ cell tumor
NUT: Nuclear protein in testis
β-hCG: β-human chorionic gonadotropin
